# Pediatric Myocarditis in a Community Pediatric Emergency Department: A Case Series

**DOI:** 10.7759/cureus.93735

**Published:** 2025-10-02

**Authors:** Tania Ahluwalia, Jillian E Nickerson, Gregory Yurasek, Elizabeth Sherwin, Jaclyn N Kline

**Affiliations:** 1 Pediatric Emergency Medicine, Children's National Hospital, Washington, DC, USA; 2 Pediatrics, Children's National Hospital, Washington, DC, USA; 3 Pediatric Cardiology, Children's National Hospital, Washington, DC, USA

**Keywords:** case series, community emergency department, emergency medicine, myocarditis, pediatrics

## Abstract

Acute fulminant myocarditis is rare but life-threatening and requires prompt recognition and intervention. Myocarditis presents variably, from nonspecific symptoms to severe arrhythmias and cardiogenic shock, complicating timely diagnosis. Community pediatric emergency departments (EDs) may be the first point of contact, necessitating high clinical suspicion among providers. This case series describes two cases of pediatric myocarditis within a six-month period at a community pediatric ED. Both cases illustrate the challenge of early recognition and the need for rapid stabilization and transfer to a tertiary care center in order to achieve favorable outcomes for these patients.

Case 1 describes a 13-year-old female who presented with nausea, vomiting, and abdominal pain, later developing complete heart block. Immediate interventions, including pharmacologic support and transcutaneous pacing, stabilized her for transfer. She was diagnosed with acute fulminant viral myocarditis, required extracorporeal membrane oxygenation (ECMO), and was ultimately discharged. Case 2 demonstrates a 10-month-old male who presented after a brief unresponsive episode, which was initially suspected to be a seizure. He developed pulseless ventricular tachycardia requiring defibrillation and was transferred after stabilization. He was confirmed to have acute fulminant viral myocarditis requiring ECMO, then ultimate placement of a ventricular assist device and prolonged intensive care hospitalization, and he is now discharged.

Both cases underscore the importance of maintaining myocarditis in the differential diagnoses for pediatric presentations that do not primarily suggest cardiac involvement. Effective use of phone consultations, shared electronic health records, and established transfer protocols facilitated timely interventions, demonstrating that community EDs can deliver high-quality care despite resource constraints. Pediatric myocarditis, though rare, necessitates rapid recognition and intervention by providers. Community pediatric EDs can significantly impact patient outcomes, highlighting the importance of preparedness for pediatric cardiac emergencies.

## Introduction

Acute cardiac conditions in children are uncommon but can present as life-threatening emergencies requiring immediate recognition and intervention [1]. Myocarditis is an inflammatory disease of the heart muscle, most commonly caused by infectious agents in children, with viruses being the predominant etiology [1,2]. Additional causes are typically classified as immune-mediated, toxic, drug-related and/or associated with systemic disorders [2]. Myocarditis poses diagnostic and management challenges due to its variable presentation, ranging from subtle nonspecific symptoms to severe arrhythmias and cardiogenic shock [3]. The incidence of pediatric myocarditis is estimated as 1 case per 100,000 children per year, making a high clinical index of suspicion critical to making this diagnosis [4]. Community pediatric emergency departments (EDs) play a critical role in identifying and stabilizing these cases, often without immediate access to advanced diagnostic tools such as echocardiography, cardiac magnetic resonance imaging, or onsite pediatric cardiologists or pediatric intensive care specialists. Despite these constraints, the availability of phone consultations with cardiology, shared electronic health data, and effective transfer protocols can enable timely and collaborative care. Consultation leveraging data-sharing by pediatric cardiologists has been shown to aid in accurate diagnosis in the newborn setting at our institution [5]. This case series highlights two presentations of pediatric myocarditis within six months at a community pediatric ED, showcasing how a high index of suspicion, prompt stabilization, and efficient teamwork can achieve favorable outcomes in these critically ill patients. In myocarditis, prompt diagnosis and access to supportive care can result in survival and possibly even complete recovery for a substantial percentage of these patients [6].

## Case presentation

Case 1

A previously healthy 13-year-old female presented to the ED with a two-day history of nausea, vomiting, and generalized abdominal pain. She had experienced over 20 episodes of non-bloody, non-bilious emesis accompanied by mild upper back pain. She denied fever, diarrhea, recent travel, trauma, or toxic exposures. Her medical history was unremarkable, with no significant family history of cardiac conditions. She had taken acetaminophen once for symptom relief and had no known allergies.

On presentation, her vital signs were unremarkable, with a heart rate of 70 beats per minute, blood pressure of 96/54 mmHg, respiratory rate of 20 breaths per minute, and oxygen saturation of 98% on room air. Physical examination revealed a soft abdomen with tenderness localized to the epigastric and left lower quadrant, without guarding, rigidity, or signs of peritoneal irritation. Her neurological and cardiovascular examinations were unremarkable. Initial laboratory studies, including a complete blood count, comprehensive metabolic panel, and urinalysis, were notable for transaminitis and findings consistent with a urinary tract infection (Table 1).

**Table 1:** Lab Results WBC: white blood cell; Hg: hemoglobin; HCT: hematocrit; PLT: platelet; Na: sodium; K: potassium; Cl: chloride; CO2: carbon dioxide; BUN: blood urea nitrogen; AST: aspartate aminotransferase; ALT: alanine aminotransferase; GGT: gamma-glutamyl transferase; RBC: red blood cell; PCR: polymerase chain reaction

Test	Case 1	Case 2	Reference Range
WBC	9.7 K/µL	12.5 K/µL	3.74-9.84 K/µL
Hg	13.2 g/dL	8.5 g/dL	11.0-14.5 g/dL
HCT	40.7%	28.3%	33.9-43.5%
PLT	384 K/µL	447 K/µL	175-332 K/µL
Neutrophils	59.30%	51%	42.5-73.2%
Na	132 mmol/L	138 mmol/L	133-143 mmol/L
K	4.7 mmol/L	4.2 mmol/L	3.3-4.7 mmol/L
Cl	103 mmol/L	106 mmol/L	97-107 mmol/L
CO2	22 mmol/L	12 mmol/L	16-25 mmol/L
BUN	30 mg/dL	10 mg/dL	6-17 mg/dL
Creatinine	1.1 mg/dL	0.28 mg/dL	0.5-1.09 mg/dL
Calcium	8.9 mg/kdL	8.9 mg/dL	9.0-10.6 mg/dL
Albumin	4.5 g/dL	3.6 g/dL	3.5-4.2 g/dL
AST	310 U/L	195 U/L	16-57 U/L
ALT	168 U/L	111 U/L	19-59 U/L
Total bilirubin	0.5 mg/dL	0.3 mg/dL	0.0-0.7 mg/dL
GGT	40 U/L	N/A	0-30 U/L
Lipase	183 U/L	N/A	10-61 U/L
Troponin	N/A	3141 ng/L	0-4 ng/L
Urine protein	1+	2+	Negative
Urine glucose	Negative	Negative	Negative
Urine ketone	Trace	1+	Negative
Urine bilirubin	Negative	Negative	Negative
Urine blood	Negative	Negative	Negative
Urine leukocyte esterase	3+	Negative	Negative
Urine nitrite	Negative	Negative	Negative
Urine WBC	31-40	3	0-3 HPF
Urine RBC	3-5	<1	0-3 HPF
Viral PCR	N/A	Adenovirus detected	Negative

Intravenous fluids, ceftriaxone, and symptomatic management, including ibuprofen and ondansetron, were given. Imaging studies, including ultrasound, were deferred pending her transfer to a tertiary care center.

**Figure 1:**
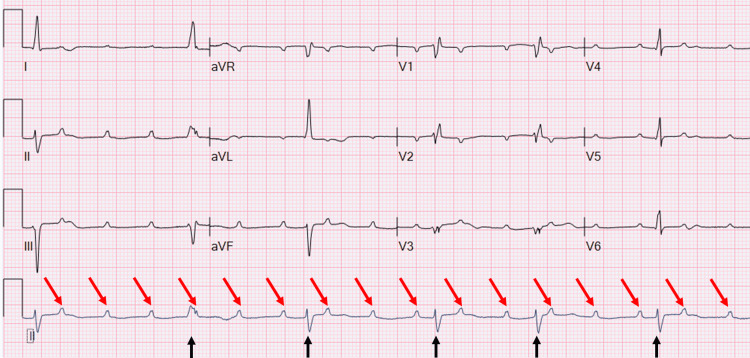
Case 1 - ECG Sinus rhythm with complete heart block. Low-voltage QRS complexes (black arrows), nonspecific T-wave abnormalities, and a prolonged QT interval (raw). Red arrows indicate non-conducted P waves.

During preparations for transfer, the patient acutely decompensated, experiencing severe bradycardia, hypotension, and oxygen desaturation. Her heart rate dropped into the 40s, her systolic blood pressure fell to 80 mmHg, and her oxygen saturation declined into the high 80s. Electrocardiogram (ECG) findings showed complete heart block (Figure 1).

Immediate interventions included administration of epinephrine and atropine, initiation of dopamine infusion, and starting transcutaneous pacing with settings titrated to maintain a rate of 70-80 beats per minute with adequate mechanical capture. These measures successfully stabilized the patient, allowing for her safe transfer to a tertiary care facility for further evaluation and management. Upon transfer to the cardiac intensive care unit (ICU), her echocardiogram showed severely depressed ventricular function. She had significant rhythm disturbance with a combination of both complete heart block with a low escape rate, as well as runs of ventricular tachycardia (VT) with a pulse. Shortly after arrival, she was intubated and placed on extracorporeal membrane oxygenation (ECMO). Later that day, she was diagnosed with acute lymphocytic myocarditis based on her endomyocardial biopsy.

**Figure 2:**
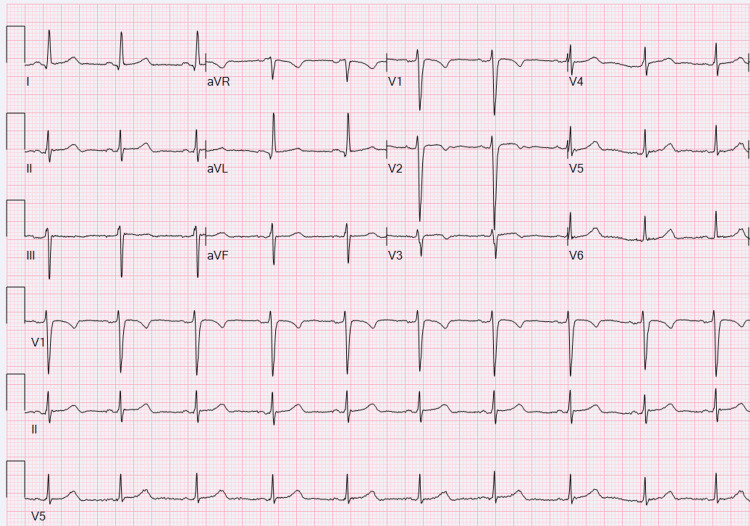
Case 1 - Follow-up ECG Normal sinus rhythm with a leftward axis and nonspecific ST-T wave changes.

Her rhythm normalized, and she was decannulated five days later, two days after that she was extubated, then transferred out of the ICU on hospital day 10, and discharged home on hospital day 15. At that time, her echocardiogram showed low-normal cardiac function. She was discharged with metoprolol for ventricular ectopy, a wearable defibrillator, and her family received cardiopulmonary resuscitation (CPR) teaching. At her follow-up appointment a month later, she reported no chest pain, palpitations, dizziness, or syncope. She continued to use her wearable defibrillator for over 23 hours per day and has had no events. She has a normal ECG (Figure 2) and Holter monitor.

Case 2

A previously healthy 10-month-old boy presented after a brief period of unresponsiveness at home. He was breastfeeding and seemed to suddenly stiffen and go limp for approximately 20 seconds. En route by ambulance, he had normal vitals, was sleepy, but was returning to baseline upon arrival to the ED per the parental report. His parents denied fever but did endorse some recent mild cough and loose stool. His most recent vaccines include his six-month immunizations, including Vaxelis, rotavirus, and PREVNAR. He did not receive vaccines for coronavirus disease 2019 or influenza.

**Figure 3:**
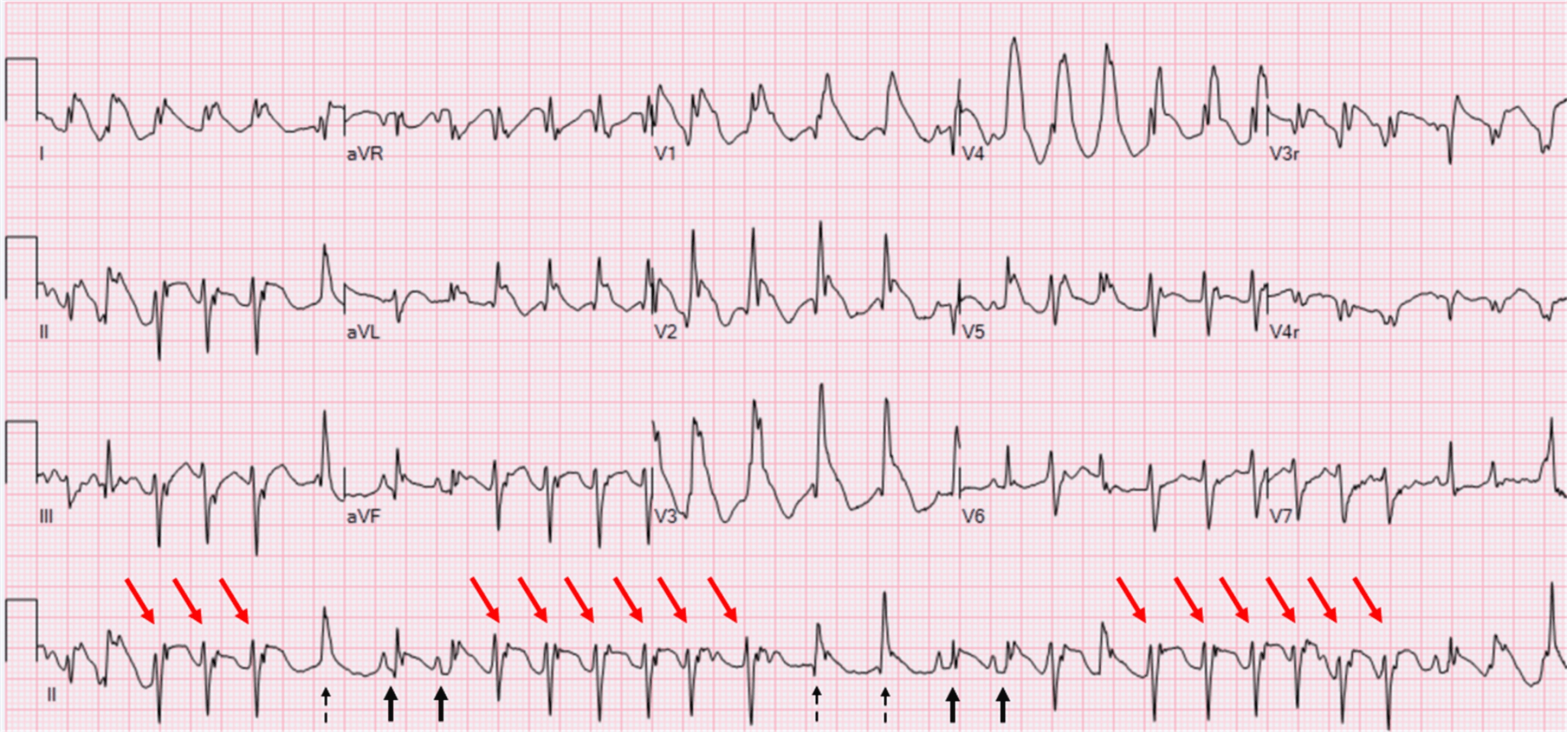
Case 2 - ECG Irregular wide QRS tachycardia. Sinus beats at 167 beats per minute (black arrows) with occasional suspected junctional or ventricular escape beats (dashed arrow). Runs of differing QRS morphology, likely nonsustained ventricular tachycardia (red arrows), at 171-220 beats per minute. ST-segment elevation noted in inferior and anterior leads.

On presentation, his vital signs were unremarkable, with a heart rate of 140 beats per minute, respiratory rate of 28 breaths per minute, blood pressure of 103/53 mmHg, and oxygen saturation of 100% on room air. An abnormal rhythm was noted on the cardiac monitor on the initial assessment, prompting an ECG documenting an irregular, wide QRS tachycardia with ST elevation in the inferior and anterior precordial leads, likely nonsustained VT at 171-220 beats per minute (Figure 3).

Cardiology was consulted and recommended urgent transfer to a tertiary care facility for further evaluation. While awaiting transfer, an intravenous line was attempted; when the tourniquet was applied, the patient had a witnessed episode of stiffening, then loss of tone and unresponsiveness. He was pulseless, and CPR was initiated; after two rounds of chest compressions, he was in pulseless VT for which defibrillation of 2 J/kg was administered. The patient regained pulses on the first pulse check after defibrillation and began to cry spontaneously. He remained in sustained VT with palpable pulses. The patient was emergently transferred to the tertiary care facility, and while awaiting air transport, he remained in VT with a pulse. With consultation from cardiology, a bolus of lidocaine 1 mg/kg was administered, with a brief return of sinus rhythm after the bolus dose; however, he then shortly returned to VT. A lidocaine infusion was started and continued en route to the tertiary care hospital.

Upon cardiac ICU admission, he was in sinus rhythm with severe ST changes and QRS widening, then had repeat VT as well as an accelerated junctional rhythm. Initial echocardiogram showed moderate to severely depressed left ventricular systolic function, moderately depressed right ventricular systolic function, mild mitral valve insufficiency, and normal coronary artery origins. He was placed on ECMO semi-electively for agitation and altered mental status. He had a prolonged cardiac ICU stay, including a second arrest in the setting of VT requiring redeployment of ECMO. He initially tested positive for adenovirus by nasopharyngeal polymerase chain reaction analysis, although endomyocardial biopsy revealed a negative immunohistochemical stain for adenovirus. Genetics was consulted, and his results were deemed non-diagnostic for his presentation. The patient was treated for presumed viral myocarditis with intravenous immunoglobulin. ECGs had persistent severe ST changes that did not resolve for 2.5 months. He had a ventricular assist device (VAD) placed 34 days after initial presentation as a bridge toward recovery. He was extubated 18 days after VAD placement. The VAD was in place for 121 days and explanted as he recovered function.

He had several electroencephalograms (EEGs), including continuous EEGs during the first two weeks of his hospitalization, and again on day 50 of hospitalization while on VAD. The first EEG showed occasional midline/right frontal sharp wave discharges and mild slowing of the background, suggesting focal cortical dysfunction, possible foci for lowered seizure threshold, in the midline/right frontal regions, likely related to a focal structural or functional disturbance. There was also mild diffuse cerebral dysfunction of nonspecific etiology. No seizures were captured during the recording. During his first two weeks, eight additional EEG reports described left temporal electrographic seizures, left frontotemporal slowing, occasional sharp wave discharges, absent awake states, absent sleep features, and mild diffuse slowing. At day 50, the EEG showed generalized slowing and a lack of normal awake features.

**Figure 4:**
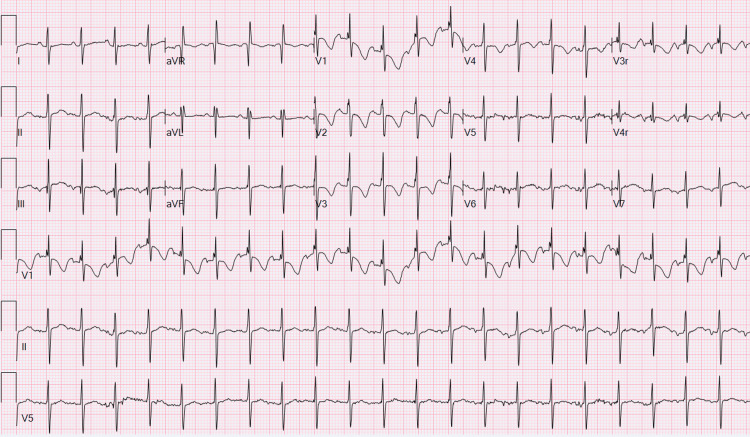
Case 2 - Follow-up ECG Normal sinus rhythm with motion artifact, right superior QRS axis, borderline corrected QT interval (QTc) of 452 ms, and nonspecific T-wave changes.

He was hospitalized for a total of 171 days, and he follows up with cardiology outpatient. His most recent ECG is shown in Figure 4.

He remains on oral heart failure medications, including Entresto, spironolactone, and furosemide. His echocardiograms show normal systolic and diastolic function. He has had no recurrent arrhythmias, and his ivabradine was weaned off recently; of note, his amiodarone had been discontinued during month 4 of hospitalization. He continues to advance nutrition, currently receiving nasogastric feeds.

## Discussion

These cases underscore the critical role of community ED providers in recognizing and managing pediatric myocarditis despite inherent challenges. Myocarditis can present with nonspecific symptoms such as fatigue, vomiting, or abdominal pain before progressing to severe and often sudden cardiovascular instability. These patients are fragile, and even minor interventions such as intravenous line placement may precipitate rapid decompensation. In the first case, the abrupt onset of bradycardia and hypotension required prompt recognition and intervention. In contrast, in the second case, the seizure was likely loss of consciousness due to arrhythmia and poor cardiac output - a sudden cardiac arrest, or possibly symptomatic ischemia. Diagnosis of fulminant myocarditis is essential as prompt supportive treatment can result in survival and possibly complete recovery of function [7-9].

Providers in this community pediatric ED demonstrated their ability to manage these complex scenarios with available resources. Phone consultations with cardiology provided timely expert guidance, ensuring accurate diagnosis and evidence-based interventions. It is known that 90% of pediatric emergency care is delivered in community EDs that care for both adults and children [10]. This specific community ED was staffed by pediatric emergency medicine physicians, pediatric nurses, and respiratory therapists, which is not a typical care model. The electronic health record is shared between this community site and the tertiary hospital, which enhances collaboration by enabling cardiologists to view ECGs remotely in real-time, facilitating rapid decision-making. However, similar information sharing is available between institutions that do not share the same electronic health records [6]. The focus on stabilization, including the use of pressors, antiarrhythmic medications, and transcutaneous pacing, highlights the preparedness and adaptability of the teams involved. The well-established transfer protocol also ensured seamless coordination with the tertiary care center, minimizing delays in accessing advanced care.

Both cases highlight the need for providers in community EDs to maintain readiness for pediatric cardiac emergencies, and the need for them to have access to pacing equipment, emergency medications, and protocols for timely consultation and transfer to higher levels of care. Pediatric readiness in community EDs is essential to ensure optimal outcomes for children presenting with life-threatening conditions like myocarditis. The presence of pediatric champions, including clinicians committed to improving pediatric care, plays a pivotal role in advocating for necessary resources, training, and protocols tailored to pediatric patients [11]. Pediatric medication dosing differs from adult care by requiring weight-based calculations to prevent under- or overdosing, highlighting the need for dosing aids, standardized protocols, and ongoing staff education. Ensuring that EDs are equipped with pediatric-specific tools, such as length-based resuscitation tapes and pediatric dosing charts, supports accurate and timely interventions. The integration of pediatric readiness principles, supported by pediatric champions, can significantly enhance the ability of community EDs to deliver high-quality emergency care to children.

Furthermore, these cases emphasize the importance of keeping a broad differential. While the initial presentation in the first case suggested a gastrointestinal issue, and the second case suggested a neurological problem, further developments revealed an underlying cardiac cause, emphasizing the complexity of diagnosing pediatric myocarditis. Because myocarditis presents in a multitude of ways and can easily be misdiagnosed as other common conditions, it is essential to maintain this diagnosis on the differential for a broad array of presentations [12]. Differential diagnoses are listed in Table 2. Red flags for myocarditis include preceding viral prodrome, arrhythmias, tachypnea, and/or tachycardia out of proportion to fever, unexplained chest pain, syncope, and/or signs of heart failure [12].

**Table 2:** Differential diagnoses for myocarditis

Category	Differential Diagnoses to Consider
Cardiac	Myocarditis
	Pericarditis
	Arrhythmias
	Hypertrophic cardiomyopathy
	Anomalous coronary artery
	Congenital heart disease
Pulmonary	Asthma exacerbation
	Pneumonia or pleuritis
	Pulmonary embolism
Infectious/Inflammatory	Sepsis
	Multisystem inflammatory syndrome in children
	Kawasaki disease
Other (Non-cardiopulmonary)	Gastroesophageal reflux disease
	Musculoskeletal pain
	Anxiety
	Anemia
	Thyroid dysfunction

While it has been traditionally thought that pediatric myocarditis presents with ECG changes, a recent case-control trial showed that over 25% of those with myocarditis had normal ECG [13]. These cases had ECGs that ranged from sinus rhythm with complete heart block to irregular wide QRS tachycardia. Both cases demonstrate how difficult it is to know not only which patients have myocarditis but also to predict who will decompensate. Studies have indicated that patients with tachycardia, tachypnea, cardiomegaly, and pericardial effusion are more likely to decompensate; however, our cases did not present with these concerning findings [14].

The strengths demonstrated by these providers in a community ED, including effective communication with consultants, timely interventions, and robust transfer protocols, highlight their ability to provide high-quality care in resource-limited settings. By leveraging available tools and focusing on collaborative care, the teams ensured positive outcomes for both patients.

Simulation exercises can enhance preparedness for rare but critical events, including pediatric myocarditis. These experiences allow teams to practice early recognition, emergency interventions, and transfer coordination, while also identifying system gaps and reinforcing pediatric-specific skills. Recent work with virtual reality (VR) simulation in pediatric cardiology has demonstrated that immersive VR scenarios, including cases on myocarditis, support interprofessional collaboration and decision-making. In this study, physician and advanced practice provider pairs reported that the VR environment was highly valuable for education [15].

## Conclusions

Pediatric myocarditis remains a complex condition to diagnose and manage due to its variable presentation and potential for rapid clinical progression. However, the above cases highlight the critical role of community pediatric ED providers in recognizing and stabilizing patients with life-threatening conditions. These cases also demonstrate how early identification, timely interventions, and close coordination with tertiary centers can lead to favorable outcomes, even in fulminant presentations. The ability of community ED teams to leverage shared electronic health records, consult subspecialists remotely, and activate transfer protocols is vital in managing pediatric cardiac emergencies. These findings underscore the importance of sustaining pediatric readiness in all emergency settings to ensure that critically ill children receive timely, high-quality care regardless of where they first present.
